# Supplementary material for: Safety of Gonadal Tissue-Derived Mesenchymal Stem Cell Therapy in Geriatric Dogs with Chronic Disease
Source: Animals (Basel). 2024 Jul 22;14(14):2134. doi: 10.3390/ani14142134 (PMC11273526; doi:10.3390/ani14142134)
Supplement: Supplementary file 1 [file animals-14-02134-s001.zip › animals-3031502-supplementary.pptx]

## Slide 1
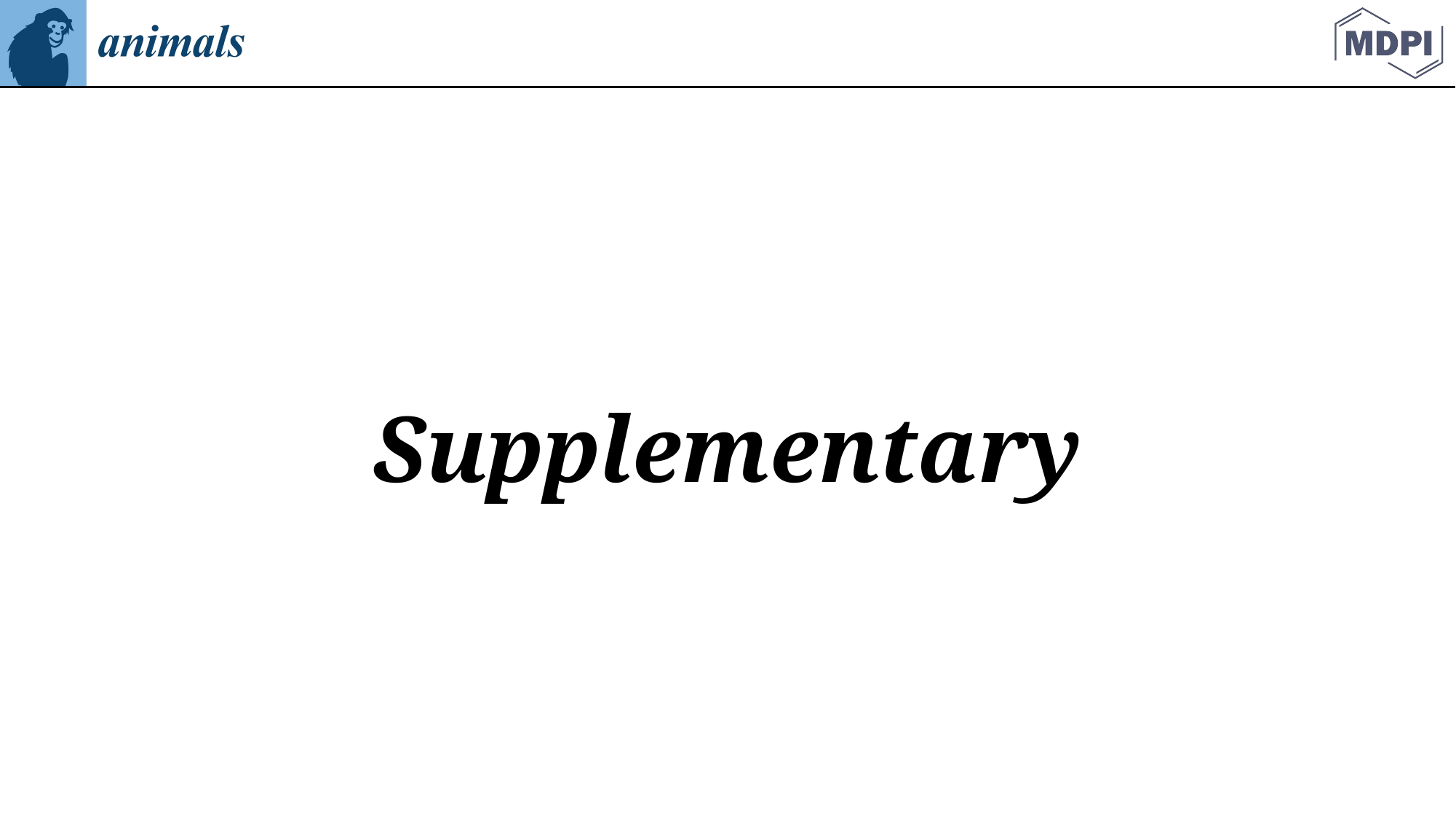

# Supplementary

## Slide 2
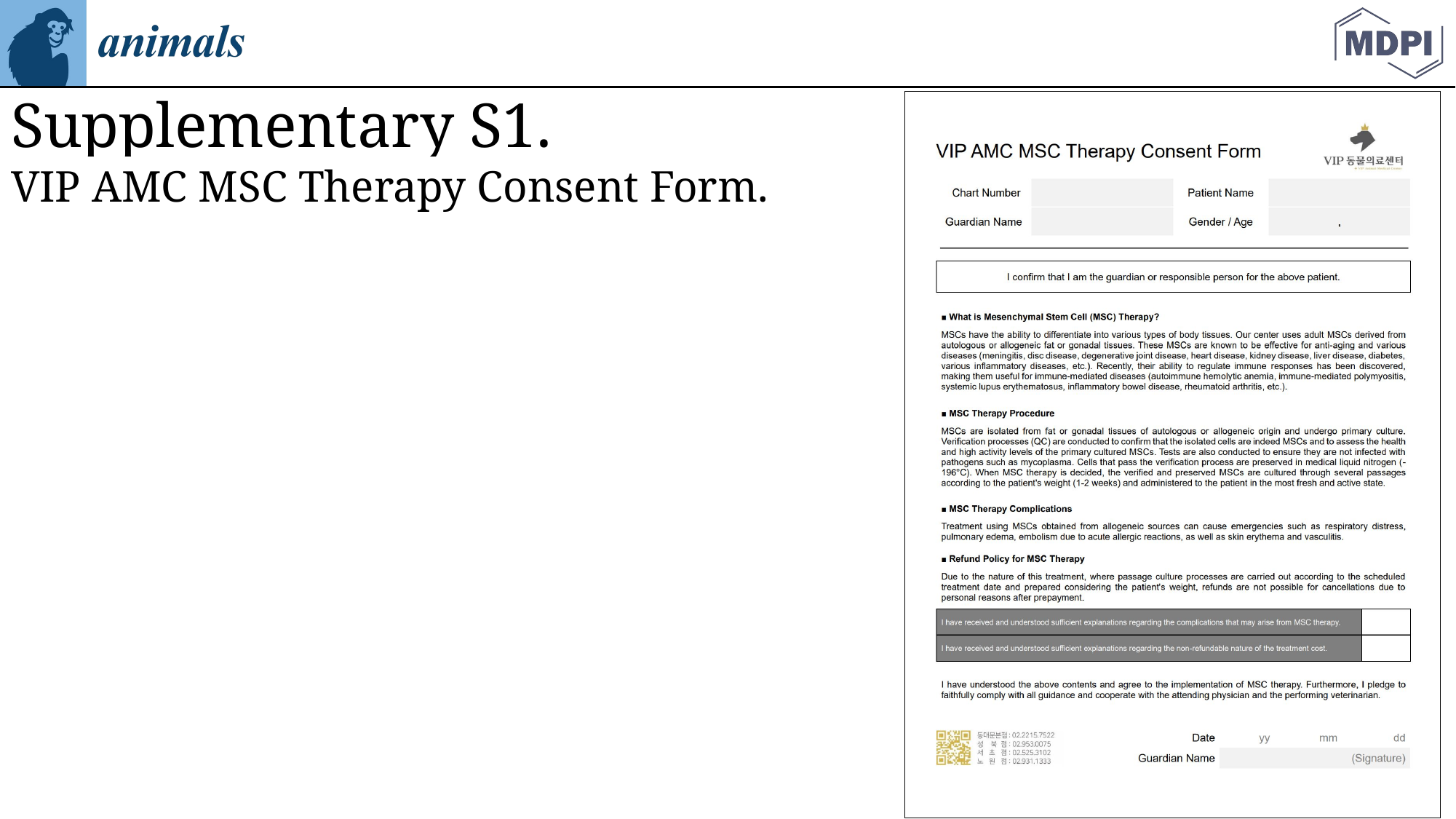

Supplementary S1.
VIP AMC MSC Therapy Consent Form.

## Slide 3
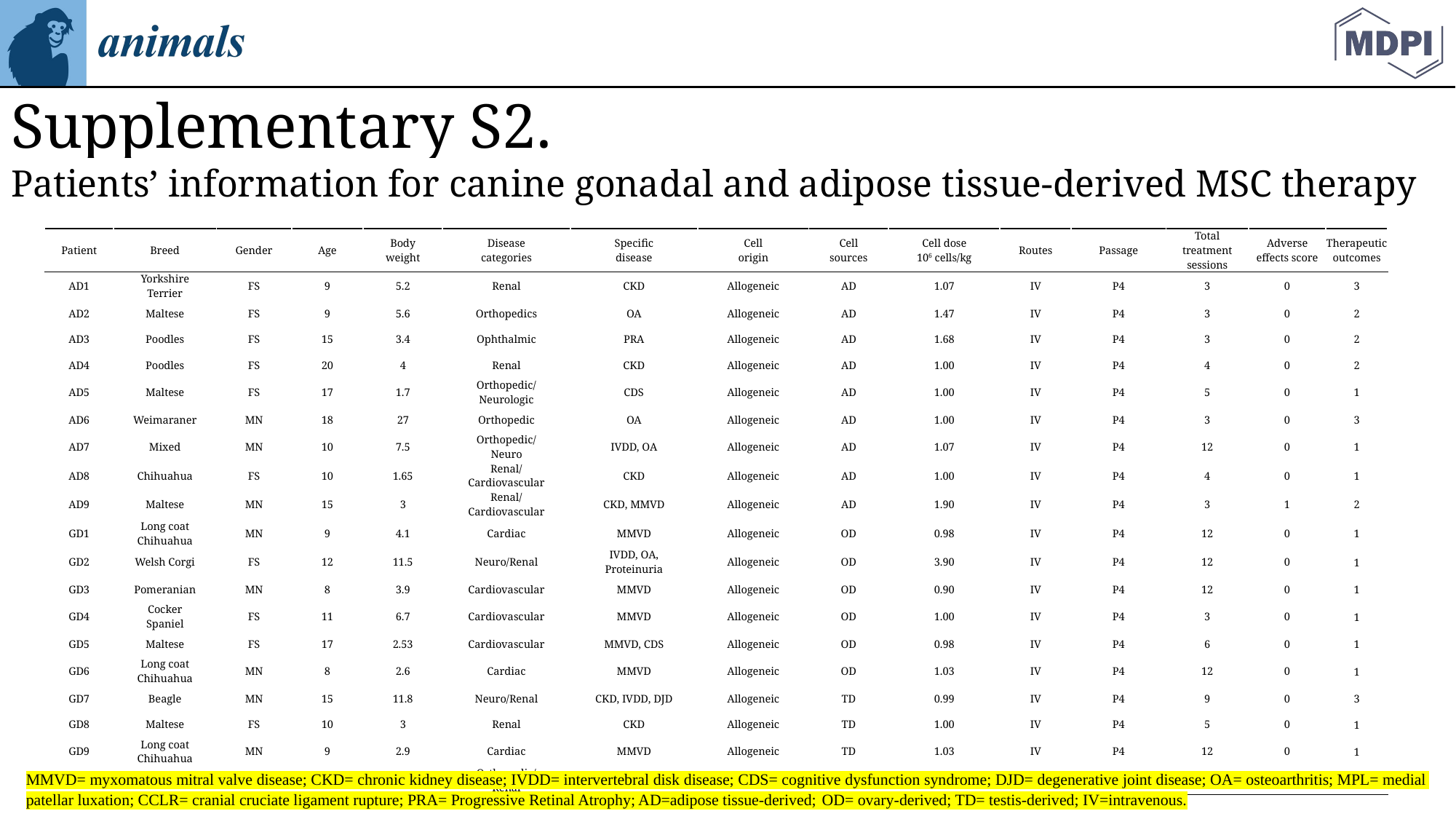

Supplementary S2.
Patients’ information for canine gonadal and adipose tissue-derived MSC therapy
| Patient | Breed | Gender | Age | Body weight | Disease categories | Specific disease | Cell origin | Cell sources | Cell dose 106 cells/kg | Routes | Passage | Total treatment sessions | Adverse effects score | Therapeutic outcomes |
| --- | --- | --- | --- | --- | --- | --- | --- | --- | --- | --- | --- | --- | --- | --- |
| AD1 | Yorkshire Terrier | FS | 9 | 5.2 | Renal | CKD | Allogeneic | AD | 1.07 | IV | P4 | 3 | 0 | 3 |
| AD2 | Maltese | FS | 9 | 5.6 | Orthopedics | OA | Allogeneic | AD | 1.47 | IV | P4 | 3 | 0 | 2 |
| AD3 | Poodles | FS | 15 | 3.4 | Ophthalmic | PRA | Allogeneic | AD | 1.68 | IV | P4 | 3 | 0 | 2 |
| AD4 | Poodles | FS | 20 | 4 | Renal | CKD | Allogeneic | AD | 1.00 | IV | P4 | 4 | 0 | 2 |
| AD5 | Maltese | FS | 17 | 1.7 | Orthopedic/ Neurologic | CDS | Allogeneic | AD | 1.00 | IV | P4 | 5 | 0 | 1 |
| AD6 | Weimaraner | MN | 18 | 27 | Orthopedic | OA | Allogeneic | AD | 1.00 | IV | P4 | 3 | 0 | 3 |
| AD7 | Mixed | MN | 10 | 7.5 | Orthopedic/ Neuro | IVDD, OA | Allogeneic | AD | 1.07 | IV | P4 | 12 | 0 | 1 |
| AD8 | Chihuahua | FS | 10 | 1.65 | Renal/ Cardiovascular | CKD | Allogeneic | AD | 1.00 | IV | P4 | 4 | 0 | 1 |
| AD9 | Maltese | MN | 15 | 3 | Renal/ Cardiovascular | CKD, MMVD | Allogeneic | AD | 1.90 | IV | P4 | 3 | 1 | 2 |
| GD1 | Long coat Chihuahua | MN | 9 | 4.1 | Cardiac | MMVD | Allogeneic | OD | 0.98 | IV | P4 | 12 | 0 | 1 |
| GD2 | Welsh Corgi | FS | 12 | 11.5 | Neuro/Renal | IVDD, OA, Proteinuria | Allogeneic | OD | 3.90 | IV | P4 | 12 | 0 | 1 |
| GD3 | Pomeranian | MN | 8 | 3.9 | Cardiovascular | MMVD | Allogeneic | OD | 0.90 | IV | P4 | 12 | 0 | 1 |
| GD4 | Cocker Spaniel | FS | 11 | 6.7 | Cardiovascular | MMVD | Allogeneic | OD | 1.00 | IV | P4 | 3 | 0 | 1 |
| GD5 | Maltese | FS | 17 | 2.53 | Cardiovascular | MMVD, CDS | Allogeneic | OD | 0.98 | IV | P4 | 6 | 0 | 1 |
| GD6 | Long coat Chihuahua | MN | 8 | 2.6 | Cardiac | MMVD | Allogeneic | OD | 1.03 | IV | P4 | 12 | 0 | 1 |
| GD7 | Beagle | MN | 15 | 11.8 | Neuro/Renal | CKD, IVDD, DJD | Allogeneic | TD | 0.99 | IV | P4 | 9 | 0 | 3 |
| GD8 | Maltese | FS | 10 | 3 | Renal | CKD | Allogeneic | TD | 1.00 | IV | P4 | 5 | 0 | 1 |
| GD9 | Long coat Chihuahua | MN | 9 | 2.9 | Cardiac | MMVD | Allogeneic | TD | 1.03 | IV | P4 | 12 | 0 | 1 |
| GD10 | Poodles | FS | 15 | 4.6 | Orthopedic/ Renal | CCLR, MPL, CKD | Allogeneic | TD | 1.00 | IV | P4 | 12 | 0 | 2 |
MMVD= myxomatous mitral valve disease; CKD= chronic kidney disease; IVDD= intervertebral disk disease; CDS= cognitive dysfunction syndrome; DJD= degenerative joint disease; OA= osteoarthritis; MPL= medial patellar luxation; CCLR= cranial cruciate ligament rupture; PRA= Progressive Retinal Atrophy; AD=adipose tissue-derived; OD= ovary-derived; TD= testis-derived; IV=intravenous.
